# Aggregation and analysis of indication-symptom relationships for drugs approved in the USA

**DOI:** 10.1007/s00228-020-02898-w

**Published:** 2020-06-03

**Authors:** Ananth Punyala, Rachana Lankapalli, Diane Hindman, Rebecca Racz

**Affiliations:** 1 Weill Cornell Medical College, New York, NY USA; 2 Division of Applied Regulatory Science, US Food and Drug Administration, Silver Spring, MD USA; 3 Emergency Department, Phoenix Children’s Hospital, Phoenix, AZ USA

**Keywords:** Text-mining, Databases, Indications, Symptoms, Natural language processing

## Abstract

**Purpose:**

Drug indications and disease symptoms often confound adverse event reports in real-world datasets, including electronic health records and reports in the FDA Adverse Event Reporting System (FAERS). A thorough, standardized set of indications and symptoms is needed to identify these confounders in such datasets for drug research and safety assessment. The aim of this study is to create a comprehensive list of drug-indication associations and disease-symptom associations using multiple resources, including existing databases and natural language processing.

**Methods:**

Drug indications for drugs approved in the USA were extracted from two databases, RxNorm and Side Effect Resource (SIDER). Symptoms for these indications were extracted from MedlinePlus and using natural language processing from PubMed abstracts.

**Results:**

A total of 1361 unique drugs, 1656 unique indications, and 2201 unique symptoms were extracted from a wide variety of MedDRA System Organ Classes. Text-mining precision was maximized at 0.65 by examining Term Frequency Inverse Document Frequency (TF-IDF) scores of the disease-symptom associations.

**Conclusion:**

The drug-indication associations and disease-symptom associations collected in this study may be useful in identifying confounders in other datasets, such as safety reports. With further refinement and additional drugs, indications, and symptoms, this dataset may become a quality resource for disease symptoms.

**Electronic supplementary material:**

The online version of this article (10.1007/s00228-020-02898-w) contains supplementary material, which is available to authorized users.

## Introduction

The last decades have vastly expanded our understanding of human disease and their treatment with medications. New advances in computational analysis and informatics approaches to investigating health care data promise to illuminate new and profound concepts for prevention, diagnosis, treatment, and care of these diseases. A large increase in FDA Adverse Event Reporting System (FAERS) reporting over the last several years may assist with earlier identification of signals [1]. New sources, such as electronic health records (EHR) and social media, can allow researchers a more comprehensive insight into patients’ lives and impact their care [2]. However, there are issues associated with analyzing these datasets. EHR and social media may be unstructured data, making it difficult to obtain large quantities of useful information for analysis [3]. In our experience, drug inefficacy may be reported as the drug’s indication or respective symptoms in FAERS or other adverse event reporting databases. These issues may lead to confounded reports and inaccurate disproportionality scores. Thus, structured medication-indication and disease-symptom relationships are imperative. Access to this information can assist clinicians in identifying the appropriate use of medications, help regulators and researchers compare drug efficacy, and aid in the analysis of drug effects relative to signs of the disease.

However, generation of these high-quality datasets may be difficult. In establishing medication-indication relationships, off-label use can be disputed or tough to document or study, and on-label indications can be difficult to extract from FDA-approved drug labels due to inconsistent labeling format and nomenclature. Additionally, disease presentations can vary across demographics, geographic locations, case severity, and disease subtypes. This can make it challenging to establish a causal relationship between certain symptoms and diseases without knowledge of the patient at hand and the actual diagnosis by physician experts.

Several existing resources cover these desired datasets to different degrees. Within the space of medication-indication relationships, the MEDication Indication (MEDI) resource [4, 5] represents an excellent aggregation and quality analysis of indications, both directly from the Side Effect Resource and RxNorm National Drug File-Reference Terminology data and from natural language processing of text resources. However, it has not been recently updated to cover newer drugs; at last update, it covered 3112 medications and 63,343 medication-indication pairs. Within the space of disease-symptom relationships, the Human Symptoms Disease Network, a substantial collection of disease-symptom relationships aggregated in part from Medical Subject Heading (MeSH) metadata found in PubMed, maximized recall of possibly pertinent disease-symptom relationships at the expense of precision [6]. This resource contains 322 symptoms and 4219 diseases, resulting in 147,978 connections. Additionally, the Disease Ontology (DO) and Human Phenotype Ontology (HPO) [7–9] have extensive disease vocabularies and descriptions. As of April 2020, DO contained over 10,000 disease IDs, and HPO contained over 13,000 terms [10, 11]. However, we have not found a single resource that integrates drug to disease to symptoms as yet.

In this paper, we aim to create a new dataset of symptoms for approved indications and common off-label uses for drugs approved in the USA. From multiple high-quality pharmacological and labeling resources, we extracted the data denoting the relationships between drug and indication and between diseases and symptoms. We believe that this dataset may assist in computational analysis of patient adverse event data. Our goal is to create a database that will allow for the identification of confounding factors in real-world data, such as FAERS, to allow for more efficient research and drug safety assessment.

## Methods

### Extraction of drug-indication relationships

**Fig. 1:**
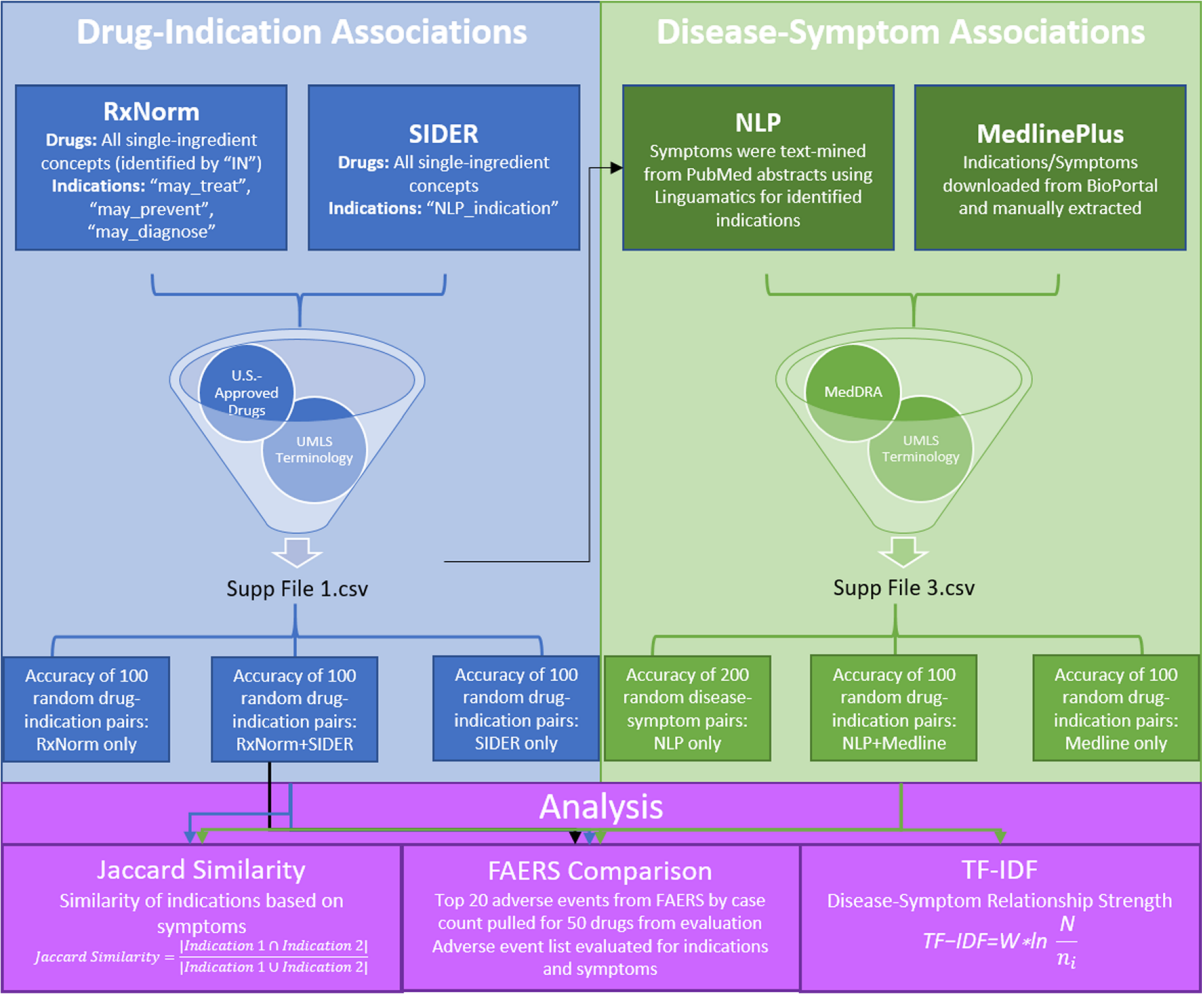
A graphical depiction of the methods and analyses used in this study

Medication-indication relationships were first compiled from RxNorm MED-RT indication information (updated April 18, 2018) and SIDER 4.1 (released October 21, 2015). RxNorm, produced by the US National Library of Medicine (NLM), is a drug nomenclature tool that groups drug source data from many sources into concepts. Each concept is represented by a Concept Unique Identifier (RxCUI) [12]. RxNorm drug concepts are linked via well-defined relationships to one another and to various external terminologies. RxNorm maps drug concept codes to disease classes from the Veterans Health Administration’s Medication Reference Terminology (MED-RT), which can then be linked to Unified Medical Language System (UMLS) Disease Concepts (US Department of Veterans Affairs, VHA). From RxNorm, all RxCUIs representing single-ingredient concepts (term type in source of “IN”) were pulled. Associated MED-RT data were used to pull any diseases or conditions linked to these drug concepts via “may_treat,” “may_prevent,” and “may_diagnose” relationships. These indication terms were then mapped to UMLS concepts via UMLS Metathesaurus Release 2018AA. The UMLS Metathesaurus is an NLM resource (Fig. 1).

The other resource, SIDER (Side Effect Resource, version 4.1 released on October 21, 2015), uses natural language processing techniques to pull MedDRA Preferred Terms (PTs, version 16.1) for indications, preconditions, and text mentions within labels approved by the US Food and Drug Administration [13, 14]. MedDRA, the Medical Dictionary for Regulatory Activities, is an international medical terminology commonly used for regulatory purposes and developed under the auspices of the International Council for Harmonisation of Technical Requirements for Pharmaceuticals for Human Use. Similar to MED-RT disease class terms, these MedDRA PTs can be linked to well-defined and comprehensive UMLS concepts. Notably, SIDER lacks coverage of biologic drugs. From SIDER 4.1, we linked generic medication names to their matching RxCUIs. We retrieved all mined terms that were linked via “NLP_indication” relationships to the medications of interest and mapped them to UMLS concepts. As SIDER includes international labels, some of the indications retrieved may not be indications approved in the USA (Fig. 1).

Finally, as both RxNorm and SIDER contain medications from other countries, we filtered for drugs approved in the USA using Drugs@FDA [15]. All extracted drug-indication relationships may be found in Supplemental File 1.

### Precision evaluation

Extracted relationships were categorized by their source and evaluated for precision. One hundred medication indications were randomly selected from both our RxNorm and SIDER 4.1 datasets. In addition, 100 medication-indication pairs were randomly selected from the intersection of the sets of RxNorm and SIDER 4.1 medication-indication pairs in order to evaluate the precision of resource combinations. We estimated the positive predictive value (precision) of each group of relationships using the results of independent manual review by the physician DH. DH was permitted to access any available resources, including but not limited to clinical literature and drug databases, in order to arrive at a judgment.

### Extraction of disease-symptom relationships

Disease-symptom relationships were extracted from MedlinePlus Health Topics pages on MedlinePlus.gov. MedlinePlus is a publicly available web resource maintained by the NLM and covering, in part, diseases, conditions, and wellness issues [16]. It covers disease-symptom relationships in free text format. MedlinePlus Health Topics entries were extracted from the 2017AA submission, released on February 2, 2017, and downloaded from BioPortal in CSV format [17]. Disease-symptom relationships were manually extracted and curated from all 2146 unique MedlinePlus Health Topics entries. Our extraction was limited to the Summary section as other sections frequently included external links to more specific resources. Using UMLS Metathesaurus Release 2017AA, disease terms were matched to their UMLS CUI, and both disease and symptom terms were converted to MedDRA Preferred Terms.

A text-mining query was developed using the natural language processing software Linguamatics I2E OnDemand. I2E OnDemand allows a user to develop queries for relationship extraction rather than keyword searching. For this study, a query was designed to text-mine Medline abstracts published between January 2009 and May 2019 for symptoms associated with the indications identified for US-approved drugs. Specifically, 100 randomly selected indications that had symptoms extracted from MedlinePlus were used as a test set to build the query. We designed the query to identify an indication (denoted by a MedDRA Preferred Term) followed in close proximity by additional MedDRA Preferred Terms (representing symptoms) with a linking term (i.e., “has symptoms”) between. Linguamatics additionally has curated synonyms for each MedDRA Preferred Term, including lower level terms and other commonly-used terms (such as “CHF” for the Preferred Term “cardiac failure congestive”); these synonyms were also included in the search. By manually analyzing literature that contained symptoms for the 100 MedlinePlus indications, it was determined that 15 words were optimal spacing for the entire ordered phrase of indication-linker-symptom(s).

Additional manual analysis identified multiple words and characters that led to false positives and therefore were negated in the query. This included the use of a comma or parentheses within the phrase, the word “with” (“indication with symptom term” usually identified a comorbidity rather than a symptom), and “secondary” (“a patient with indication…history of…symptom term” did not consistently lead to a direct indication-symptom relationship). A full list of linking and negated terms may be found in Supplemental File 2.

Upon completion of the query, all indications identified in SIDER and RxNorm were queried for symptoms reported in Medline abstracts. All extracted disease-symptom relationships may be found in Supplemental File 3.

### Precision evaluation

Extracted relationships were categorized by their source and evaluated for precision. One hundred disease-symptom pairs were randomly selected from our MedlinePlus dataset, distinct from those used to build the text-mining query. In addition, 200 disease-symptom pairs were randomly selected from our text-mined dataset. Finally, 100 disease-symptom pairs were randomly selected from the intersection of the sets of MedlinePlus and natural language processing (NLP) disease-symptom pairs in order to evaluate the precision of resource combinations. We estimated the positive predictive value (precision) of each group of relationships using the results of independent manual review by the physician RL. RL was permitted to access any available resources, including but not limited to clinical literature and drug databases, in order to arrive at a judgment.

### Precision evaluation of drug-indication-symptom associations

Drug-indication and disease-symptom relationships were concatenated. To evaluate the precision of these linked relationships, 100 drug-indication-symptom relationships, irrespective of source, were randomly selected for manual review by the physician RL. RL was permitted to access any available resources, including but not limited to clinical literature and drug databases, in order to arrive at a judgment. Individual relationships (drug-indication and disease-symptom) were evaluated, but the entire association was determined to be a true positive only if both associations were correct.

### Evaluation of relationship strength

To evaluate the strength of the association between disease and symptom, a modified Term Frequency Inverse Document Frequency (TF-IDF) score was calculated [6]:

$$ \mathrm{TF}-\mathrm{IDF}=\mathrm{W}\ast \ln \frac{\mathrm{N}}{n_{\mathrm{i}}} $$

where N is the number of diseases with symptoms in the disease-symptom dataset curated from NLP and MedlinePlus, n_i_ is the number of diseases with the symptom of interest, and W is the number of times the symptom and disease co-occurred in text-mining and MedlinePlus (absolute co-occurrence). The higher the resulting TF-IDF score, the stronger the relationship between indication and symptom. All TF-IDF scores may be found in Supplemental File 3.

### Similarity evaluation between indications

Diseases that are treated with the same drug may share targets in their pathogenesis, which may result in shared symptoms. To identify if diseases that are treated by the same drug share symptoms, indications were grouped by drugs, and the similarity between each group was calculated using the Jaccard similarity calculation. The similarity was computed as follows:

$$ Jaccard\ Similarity\ \left( Ind\ 1, Ind\ 2\right)=\frac{\left( number\ of\ symptoms\ shared\  by\  Ind\ 1\  and\  Ind\ 2\right)}{\left( number\ of\ symptoms\ associated\ with\ either\  Ind\ 1\  or\  Ind\ 2\right)} $$

### Identification of indications and symptoms in FAERS data

To identify indications and symptoms discovered by this analysis that may be found in FAERS data, 50 random drugs were selected from the drugs that were evaluated for precision. The top 20 adverse events by case count with a Proportional Reporting Ratio (PRR) greater than 2.0 in FAERS for each drug were selected for analysis. This PRR cutoff was selected to represent adverse events that were reported twice as frequently for the drug of interest compared to other drugs in FAERS. The list of the top 20 reported adverse events for each drug was crosschecked against the drug’s indications as well as symptom terms for those indications that had a TF-IDF score above our threshold, which was set to maximize precision of the accepted disease-symptom pairs.

## Results

**Fig. 2:**
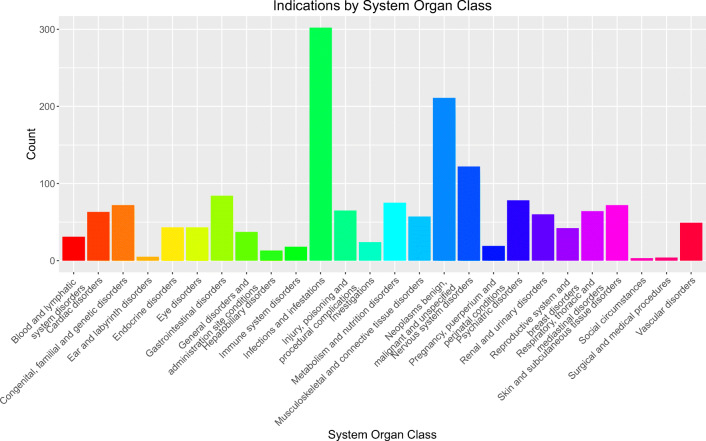
Distribution of US drug approvals by source and year. The majority of drugs included in this dataset were approved post-1982, according to FDA Orange Book data. Most approval dates pre-1982 are listed as “Approved Prior to Jan 1, 1982” in the Orange Book. Most of the drugs extracted after 2013 were retrieved from RxNorm

From SIDER and RxNorm combined, a total of 1361 US-approved drugs were pulled. This includes 867 drugs from SIDER and 1328 drugs from RxNorm. When compared with the FDA’s Orange Book, a resource containing approved drugs and their therapeutic equivalents, 86 single ingredient drugs were missing from either source and thus from our dataset. Many of the missing drugs were single-use, single-ingredient drugs (such as diagnostic agents); a complete list can be found in Supplemental File 4. Finally, the drugs in the combined RxNorm and SIDER dataset were approved across many decades, with the earliest approval being 1960 and the latest approval being 2018 (Fig. 2). While SIDER was last updated in 2015, some US drug approvals post-2015 were still included as they were approved in other countries before the last update.

From SIDER and RxNorm combined, a total of 1656 indications were extracted along with a total of 8144 drug-indication associations. Indications were counted by UMLS CUI that was extracted or converted from the source terminology without de-duplication for similar indications (i.e., both “anxiety” and “anxiety disorder” were reported as separate indications, but the drugs treating these respective indications may be treating the same disease in theory). This includes 3063 unique drug-indication associations from SIDER, 4095 unique drug-indication associations from RxNorm, and 986 drug-indication associations found in both sources. These indications covered a wide variety of MedDRA System Organ Classes (Supplemental File 5, Fig. 1).

Additionally, 430 diseases were extracted from MedlinePlus as unmapped free text. Once MedlinePlus diseases were converted to UMLS CUIs, 253 diseases from MedlinePlus mapped directly to indications from the combined SIDER and RxNorm dataset. This left 177 MedlinePlus diseases that did not map to an indication and 1403 indications from RxNorm/SIDER without symptoms from MedlinePlus. All indications from RxNorm and SIDER were input into the text-mining algorithm, but only 923 (56%) indications returned symptom results. A total of 41 MedlinePlus diseases did not return results in the text-mining query, bringing the total to 964 unique diseases with one or more symptoms in our dataset. A total of 2201 unique symptoms were collected between MedlinePlus and text-mining. On average, indications with reported symptoms had a median of 5 symptoms, with one being the fewest number of symptoms and 316 (infection) being the greatest number of reported symptoms (Supplemental File 5, Fig. 2). Combining MedlinePlus and the text-mining algorithm, a total of 692 indications reported by RxNorm and SIDER remained unmapped to symptoms. TF-IDF scores ranged from 2.15 to 137.45, with a median of 4.93.

Upon evaluation, our drug to indication sources, SIDER and RxNorm, were found to be relatively correct according to a physician evaluation. Of the 100 drug-indication associations from SIDER evaluated, 24 were not correct (76% precision), and of the 100 drug-indication associations from RxNorm evaluated, 14 were not correct (86% precision). Additionally, 100 drug-indication associations that were found in both resources resulted in only one association that was not correct (99% precision) (Table 1).

**Table 1:** Precision of dataset associations

Source	Number of pairs	Precision	Estimated number of false positives
Drug-indication associations			
SIDER	3063	76%	735
RxNorm	4095	86%	573
SIDER + RxNorm	986	99%	10
Disease-symptom associations			
MedlinePlus	1317	98%	26
NLP	8901	57%	3827
MedlinePlus + NLP	105	99%	1
Drug-indication-symptom associations			
All	149,164	58%	62,649

**Fig. 3:**
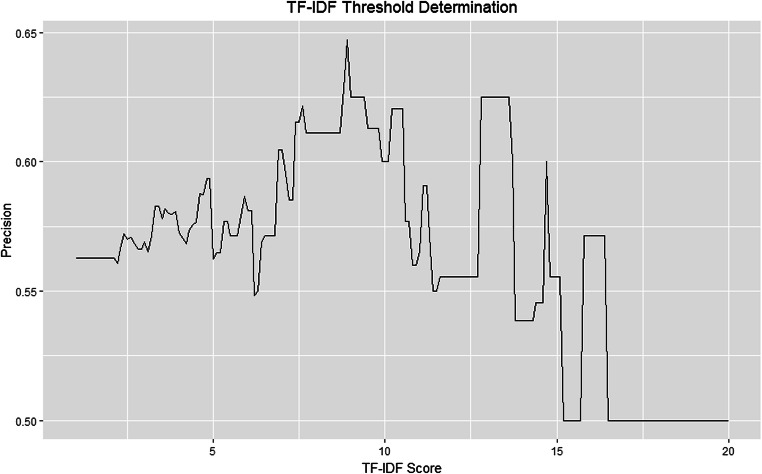
Threshold determination of TF-IDF scores based on precision. When TF-IDF scores were used as a threshold, precision ranged from 0.54 to 0.65

For the disease-symptom associations, Medline and the text-mining query were evaluated separately. Of the 100 disease-symptom associations from Medline evaluated, two associations were not accurate (98% precision). Of the 200 disease-symptom associations from the text-mining query evaluated, 87 were not accurate (57% precision). Of the 100 disease-symptom relationships that were found in both resources, one was not accurate (99% precision) (Table 1). From this evaluation, we developed a TF-IDF score threshold to maximize precision. Any associations found to have a TF-IDF score above 8.95 maximized the precision of the text-mining query at 0.65 (Fig. 3). At this threshold, 90 true positives from the precision analysis were rejected, and 75 false positives were rejected.

Finally, from the drug-indication-symptom evaluation, 42 associations were not accurate (58% precision); from these, seven were not accurate based on the drug-indication relationship, 29 were not accurate based on the disease-symptom relationship, and six were not accurate based on both the drug-indication and disease-symptom relationship (Table 1).

We hypothesized that indications treated by the same drug may share overlapping symptoms, as these diseases may share pathophysiology and involve the same receptor classes (serotonergic, adrenergic) for drug targeting. To evaluate this hypothesis, we assessed the symptom similarity between diseases sharing the same treatment compared with random diseases. For all diseases against each other, the average similarity was 0.006. The average similarity for diseases against other diseases being treated by the same drug was 0.024 (Supplemental File 5, Fig. 3). While slightly more similar on average, our dataset does not demonstrate that indications with shared drug treatment also share symptoms.

To evaluate the utility of this dataset in identifying indications and symptoms in other datasets that may be used for research or drug safety assessment, we evaluated the top twenty reported adverse events in FAERS for 50 drugs. We found that 36 drugs (72%) had at least one reported indication in the top 20 reported adverse events and 30 drugs (60%) had at least one reported symptom in the top 20 reported adverse events. For example, celecoxib was associated with 11 indications in our dataset, the majority of which were related to pain or arthritis. Of the top 20 adverse events reported in FAERS for celecoxib, three were reported as indications in our dataset (pain, arthritis, and osteoarthritis). Additionally, for the 11 indications, we evaluated all symptoms above the TF-IDF cutoff of 8.95 and found that one (arthralgia) was also reported in the top 20 adverse events for celecoxib. This analysis reveals an important limitation of FAERS and a key reason why a dataset such as the one created here is necessary.

## Discussion

In this study, we identified drug-indication relationships for US-approved drugs using two sources, SIDER and RxNorm. We identified symptoms for 58% of these indications using a combination of manual extraction from MedlinePlus and text-mining PubMed abstracts. We further computed the strength of these indication-symptom relationships by calculating a TF-IDF score and found after an evaluation of 200 indications that a TF-IDF threshold of 8.95 maximized our symptom precision at 0.65. Finally, an evaluation of the utility of this dataset in identifying confounding indications and symptoms in FAERS found that 72% of a drug subset had at least one indication in the top 20 adverse events and 60% of drugs had at least one symptom of one of their indications. In summary, we have compiled a dataset of symptoms for approved and common off-label uses of drugs approved in the USA that may be used for drug safety assessment and research.

There are many potential applications for this dataset. We have demonstrated that FAERS is confounded by indications and symptoms and this dataset may have a utility in identifying such confounders. Indication and symptom confounders may affect research using datasets like FAERS, such as adverse event prediction. We have previously developed a model to make predictions for multiple adverse events across drug classes using FAERS and label data, but several false-positive predictions resulted from symptoms and indications reported in FAERS data [18]. When the model was re-run and the indications and symptoms in our dataset were removed from the input dataset, performance slightly improved (unpublished data). Similarly, this dataset may aid in identifying potential confounders in clinical trials, EHR, and other real-world evidence datasets. This information is often considered for research and drug safety assessment; flagging or removing confounders, such as indications and symptoms, may make for a cleaner dataset to identify safety signals.

However, in utilizing this dataset for research and other investigations of real-world datasets, caution must be employed. Complete removal of indications and/or symptoms may result in removal of true adverse event signals. Drugs could potentially worsen comorbid conditions or symptoms that are included in this dataset, and therefore removal of these terms may result in missed signals. Disease presentations also need not include all the symptoms included in a dataset of disease-symptom relationships. When incorporating this set or other similar sets into drug safety research, one should weigh the risks and benefits of removing indications and symptoms automatically. In many cases, identification or flagging of suspected confounders could retain sufficient human oversight.

Compilation of this dataset was limited by several factors, including the sources. In this initial study, we focused on text-mining for symptoms related to primarily on-label indications. By adding additional sources for symptoms and indications, particularly off-label indications, a more robust data source may be built that can be used for multiple purposes. Additionally, inclusion of more symptom data, such as manually extracted data, may help account for automated text-mining errors. Finally, we were limited by text-mining only the abstracts. While abstracts may collect the most common symptoms, we may miss symptoms specific to certain disease states or populations. Our precision evaluation did not capture symptoms that may be missing from our dataset (false negatives), which may have limited our analysis to determine similarity of indications with shared drug treatment. Additional sources, including symptoms from the full-text, additional years, or other manually curated sources, may alleviate this limitation.

We are currently exploring new expansions and developments to the dataset. First, addition of other data sources, particularly off-label indications, will be important to capture. We have been evaluating additional sources of indications, such as FAERS, manual extraction of labeled indications, and curated databases. Also, to increase precision and quality for the symptom relationships, sentence mapping and grammatical processing could be employed; this would allow specific relationships to be extracted based upon sentence structure that would more closely approximate semantic meaning. Additionally, comorbidity data and concurrent medications may be incorporated into the resource to further identify confounders in datasets like FAERS. We are furthermore evaluating our ability to add mappings to other UMLS terminologies to make the dataset more universal. Finally, we would like to transform the dataset into an ontology format, which will make it more machine readable than the current relational database format. As we continue to explore new expansions and developments to the dataset, our aim is to increase the precision of the dataset. With additional precision, this dataset may be appropriate for use in automated removal of indications and symptoms.

## Conclusion

In conclusion, we have created a dataset of symptoms for approved indications and common off-label uses of approved US drugs, with the aim to create a comprehensive knowledge base with multiple uses in research and drug safety assessment. These mappings allow for data cleaning and confounder identification. To our knowledge, this is the first attempt to text-mine symptoms for indications of all US drugs. With additional expansions and developments, including off-label indications and ontology creation, this dataset has potential to become a high-quality source for disease symptoms.

## Electronic supplementary material

ESM 1 (CSV 322 kb).

ESM 2 (PDF 67 kb).

ESM 3 (CSV 538 kb).

ESM 4 (CSV 1 kb).

ESM 5 (PDF 175 kb).

## Data Availability

All data generated or analyzed during this study are included in this published article (and its supplementary information files).

## Glossary

CUI: Concept Unique Identifier
EHR: Electronic health records
FAERS: FDA Adverse Event Reporting System
FDA: Food and Drug Administration
MED-RT: Medication Reference Terminology
NLM: National Library of Medicine
PT: Preferred Term
SIDER: Side Effect Resource
TF-IDF: Term Frequency Inverse Document Frequency
UMLS: Unified Medical Language System
