# Supplementary material for: Aggregation and analysis of indication-symptom relationships for drugs approved in the USA
Source: Eur J Clin Pharmacol. 2020 Jun 3;76(9):1291–9. doi: 10.1007/s00228-020-02898-w (PMC7419351; doi:10.1007/s00228-020-02898-w)
Supplement: Supplementary file 2 — (PDF 67 kb). [file 228_2020_2898_MOESM2_ESM.pdf]

<u>“Linker” words</u>

Symptom
Exhibit
Present
Trigger
Develop

<u>Negated words and characters between indication and symptom list due to high false positive rate</u>

,
(
who
and
with
of
in
during
on
by
measure
with
as
due
after
previous
secondary
from
than
suspicion
non
chemotherapy
was
to
history
score
all animals via an ontology (e.g. mouse, rat, rabbit)
