# Supplementary figures and images for: Aggregation and analysis of indication-symptom relationships for drugs approved in the USA
Source: Eur J Clin Pharmacol. 2020 Jun 3;76(9):1291–9. doi: 10.1007/s00228-020-02898-w (PMC7419351; doi:10.1007/s00228-020-02898-w)

Figure 1

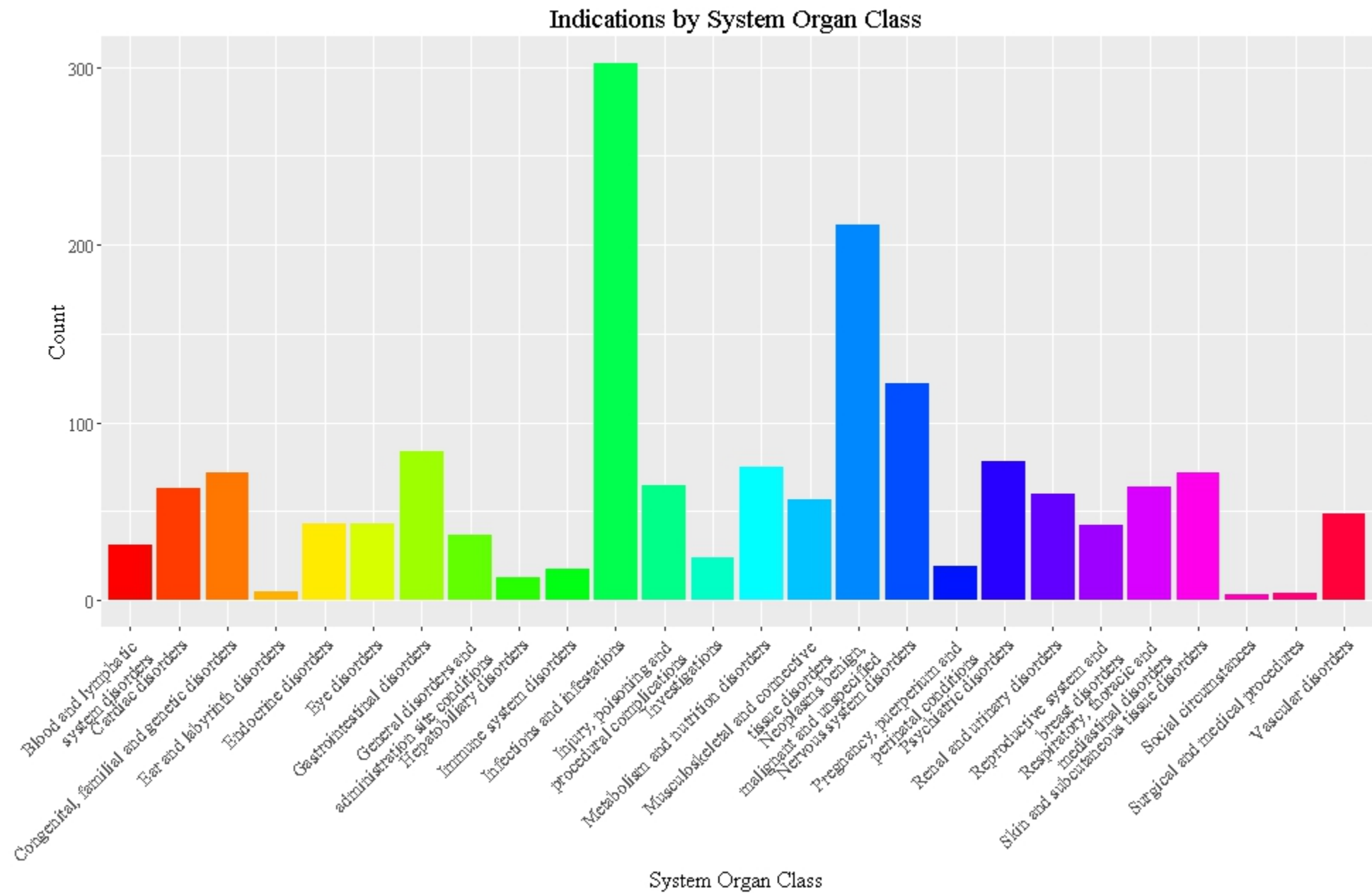

Figure 2

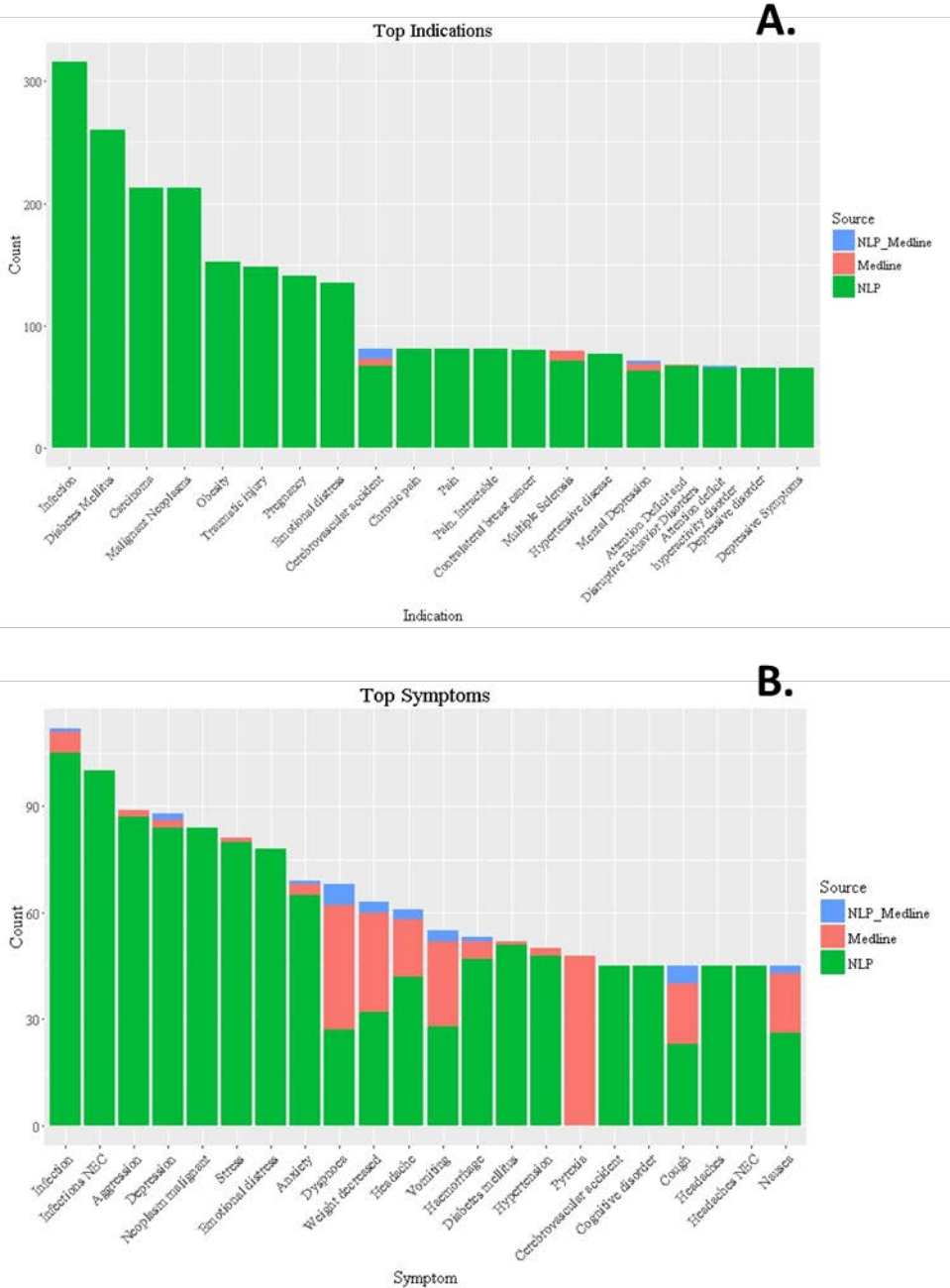

Figure 3

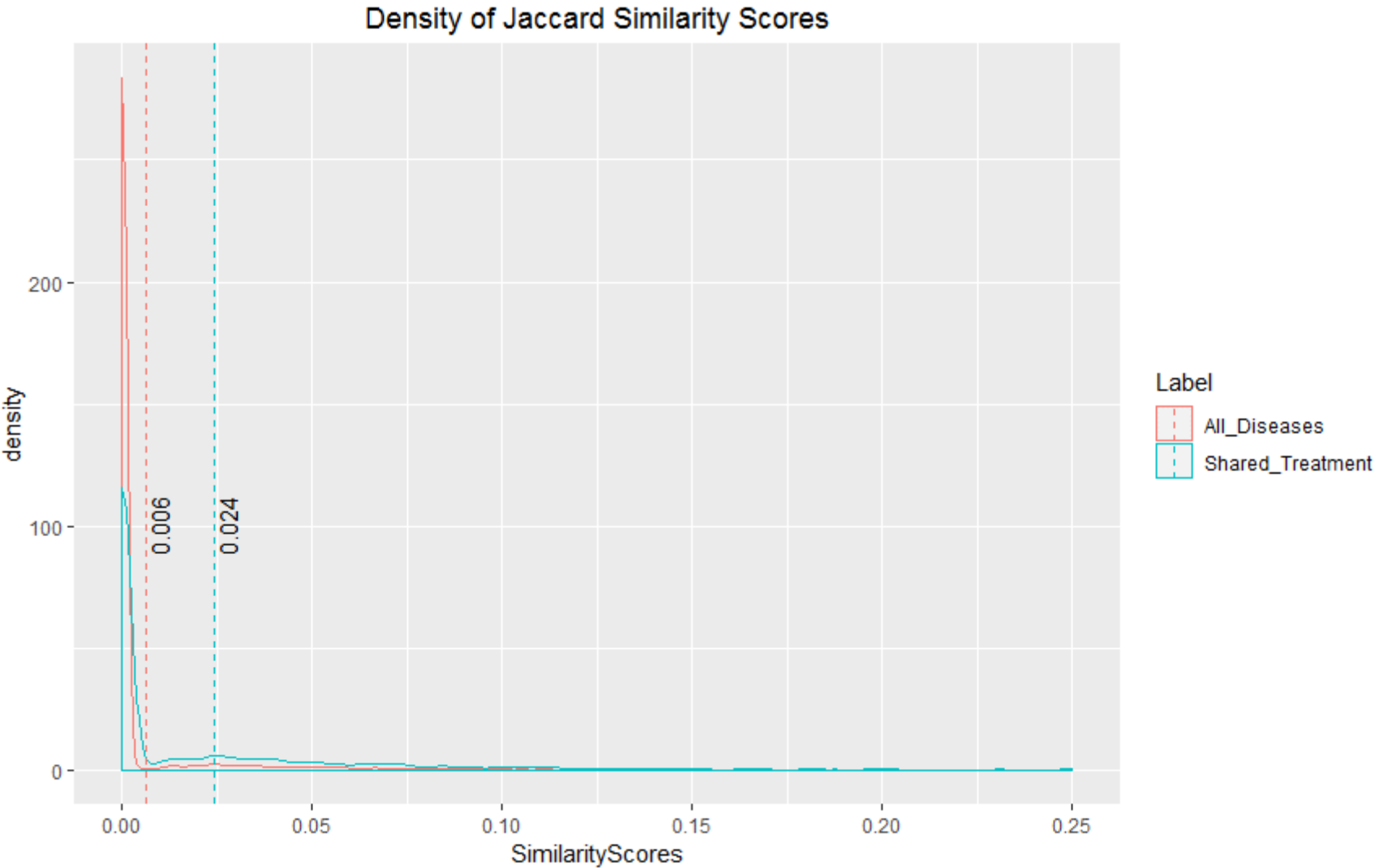

Supplement: Supplementary file 5 — (PDF 175 kb). [file 228_2020_2898_MOESM5_ESM.pdf]
